# Tear Lysozyme in Sjögren´s syndrome, Meibomian gland dysfunction, and non-dry-eye

**DOI:** 10.5935/0004-2749.20220017

**Published:** 2025-08-21

**Authors:** Martín Berra, Gustavo Galperín, Federico Berra, Maria Isabel Marquez, Mariana Mandaradoni, Julia Tau, Alejandro Berra

**Affiliations:** 1 Hospital Oftalmológico “Pedro Lagleyze”, Buenos Aires, Argentina; 2 Bioquímica Ocular, Buenos Aires, Argentina; 3 Hospital Posadas Buenos Aires, Argentina; 4 Laboratorio Traslacional de Inmunopatologia & Oftalmologia, Departamento de Patologia, Facultad de Medicina, Universidad de Buenos Aires, Buenos Aires, Argentina

**Keywords:** Lysozyme, Sjogren’s syndrome, Meibomian gland dysfunction, Non-dry-eye, Lisozima, Síndrome de Sjögren, Disfunção da glândula tarsal, Olho não seco

## Abstract

**Purpose:**

To evaluate the concentration of tear lysozyme in individuals with Sjogren´s syndrome, meibomian gland dysfunction, and non-dry-eye disease.

**Methods:**

Ninety subjects were recruited for this study, including 30 with Sjogren´s syndrome, 30 with meibomian gland dysfunction, and 30 with non-dry-eye disease. All subjects were referred to participate in the study based on a “dry eye” investigation. They underwent a complete ocular surface ophthalmic examination encompassing ocular surface disease index, biomicroscopy, tear break-up time, Schirmer test type I, conjunctival vital staining with fluorescein and lissamine green, tear lysozyme concentration, and impression cytology.

**Results:**

Clinical tests yielded the following results: ocular surface disease index Sjogren´s syndrome: 64.5 ± 22.6 meibomian gland dysfunction: 43.5 ± 21.4, non-dry-eye disease: 6.7 ± 4.3 (p=0.02 between groups); Schirmer I test (mm/5 min): Sjogren´s syndrome: 4.95 ± 2.25, meibomian gland dysfunction: 13.28 ± 1.53, non-dry-eye disease 13.70 ± 1.39 (*p<*0.01 Sjogren´s syndrome vs. non-dry-eye disease and p<0.01 meibomian gland dysfunction vs. non-dry-eye disease); tear break-up time (seconds): Sjogren´s syndrome: 3.97 ± 1.47, meibomian gland dysfunction: 3.95 ± 0.86, non-dry-eye disease: 7.25 ± 1.90 (p<0.01 Sjogren´s syndrome vs. non-dry-eye disease and p<0.01 meibomian gland dysfunction vs. non-dry-eye disease); Lissamine green score: Sjogren´s syndrome-dry-eye: 6.18 ± 2.14, meibomian gland dysfunction-dry-eye: 5.27 ± 1.27, non-dry-eye disease: 1.52 ± 0.97 (p<0.01 Sjogren´s syndrome vs. non-dry-eye disease and p<0.01 meibomian gland dysfunction vs. non-dry-eye disease); impression cytology score: Sjogren´s syndrome: 1.88 ± 0.92, meibomian gland dysfunction: 1.67 ± 0.56, non-dry-eye: 0.45 ± 0.44 (p<0.01 Sjogren´s syndrome vs. non-dry-eye disease and p<0.01 meibomian gland dysfunction vs. non-dry-eye disease) and; tear lysozyme concentration (mg/mL): Sjogren´s syndrome: 751.25 ± 244.73, meibomian gland dysfunction: 1423.67 ± 182.75, non-dry-eye disease: 1409.90 ± 188.21 (p<0.01 Sjogren´s syndrome vs. non-dry-eye disease and p<0.01 Sjogren´s syndrome vs. meibomian gland dysfunction).

**Conclusion:**

The concentration of lysozyme in the tears was lower in Sjögren’s syndrome patients than in meibomian gland dysfunction and non-dry-eye disease groups. Hence, the lacrimal lysozyme could be considered as a simple, non-invasive, and economical biomarker to differentiate between Sjögren’s syndrome dry eye disease and meibomian gland dysfunction dry eye disease.

## Abstract (pt)

**Objetivo:**

Avaliar a concentração de lisozima lacrimal na síndrome de Sjögren, disfunção da glândula meibomiana e doença ocular não seca.

**Métodos:**

Noventa indivíduos foram recrutados para este estudo: 30 indivíduos com síndrome de Sjögren, 30 indivíduos com disfunção da glândula meibomiana e 30 indivíduos com doenças oculares não secas. Todos os sujeitos foram encaminhados para estudo de investigação de “olho seco”, sendo submetidos a um exame oftálmico completo de superfície ocular (índice de doença da superfície ocular, biomicroscopia, tempo de ruptura do rasgo, teste de Schirmer tipo I, coloração vital da conjuntiva com fluoresceína e lissamina verde e concentração e citologia da lisozima lacrimal.

**Resultados:**

Os testes clínicos mostraram: índice de doença da superfície ocular e Síndrome de Sjögren: 64,5 ± 22,6, disfunção da glândula meibomiana: 43,5 ± 21,4, doença ocular não seca: 6,7 ± 4,3 (valor de p=0,02 entre grupos); Teste de Schirmer I (mm/5min), síndrome de Sjögren: 4,95 ± 2,25, disfunção da glândula meibomiana: 13,28 ± 1,53, doença ocular não seca: 13,70 ± 1,39 (p<0,01, síndrome de Sjögren vs. doença ocular não seca e p<0,01, disfunção da glândula meibomiana vs. doença ocular não seca); tempo de ruptura do rasgo (segundos), síndrome de Sjögren: 3,97 ± 1,47, disfunção da glândula meibomiana: 3,95 ± 0,86, doença ocular não seca: 7,25 ± 1,90 (p<0,01, síndrome de Sjögren vs. doença ocular não seca e p<0,01, disfunção da glândula meibomiana vs. doença ocular não seca); escore de lissamina verde, síndrome de Sjögren - olho seco: 6,18 ± 2,14, disfunção da glândula meibomiana - olho seco: 5.27 ± 1,27, doença ocular não seca: 1,52 ± 0,97 (p<0,01, síndrome de Sjögren vs. doença ocular não seca e p<0,01, disfunção da glândula meibomiana vs. doença ocular não seca); escore de citologia de impressão, síndrome de Sjögren: 1,88 ± 0,92, disfunção da glândula meibomiana: 1,67 ± 0,56, doença ocular não seca: 0,45 ± 0,44 (p<0,01, síndrome de Sjögren vs. doença ocular não seca e p<0,01, disfunção da glândula meibomiana vs. doença ocular não seca) e concentração de lisozima lacrimal (mg/mL), síndrome de Sjögren: 751,25 ± 244,73, disfunção da glândula meibomiana: 1423,67 ± 182,75, doença ocular não seca: 1409,90 ± 188,21 (p<0,01, síndrome de Sjögren vs. doença ocular não seca e p<0,01, síndrome de Sjögren vs. disfunção da glândula meibomiana). Conclusão: A concentração de lisozima nas lágrimas foi menor nos pacientes com síndrome de Sjögren do que nos grupos com disfunção da glândula meibomiana e doença ocular não seca. A lisozima lacrimal poderia ser considerada como um biomarcador simples, não invasivo e econômico para diferenciar o olho seco da síndrome de Sjögren do olho seco da disfunção da glândula meibomiana.

## INTRODUCTION

Dry eye disease (DED) is defined as a multifactorial disease of the ocular surface and is characterized by the loss of homeostasis of the tear film. This condition is accompanied by ocular symptoms in which tear film instability and hyperosmolarity, ocular surface inflammation and damage, and neurosensory abnormalities play etiological roles^(1)^.

Burning, stinging, tearing, and itching are the typical symptoms experienced by the patients^(1)^. If severe enough, they can cause discomfort and affect the quality of life and work productivity^(2)^ .

The prevalence of DED has been reported to range from 5.5% to 33.7% in many studies^(3,4)^. In the United States, the economic burden of DED to the society is calculated to be $55.4 billion. An average DED patient is estimated to spend approximately $783 annually for managing the condition^(5)^.

The dry eye etiologies are divided into two predominant and non-mutually exclusive subgroups: aqueous deficient dry eye disease (ADDE) and evaporative dry eye (EDE). ADDE pertains to conditions affecting the lacrimal gland function and could be further classified into two subtypes: Sjögren´s syndrome (SS) dry eye and non-Sjögren´s syndrome dry eye^(1)^. EDE is thought to include both lid-related (for example, meibomian gland dysfunction (MGD) and blink-related) and ocular surface-related causes^(1)^. MGD is considered to be the leading cause of dry eye in clinical and population-based studies^(1)^.

Sjögren’s syndrome (SS) is a multisystem autoimmune disease characterized by T-cell infiltration and B-cell hyperactivity in lacrimal and salivary glands, which lead to fibrosis and progressive destruction of the tissues^(6)^. SS is one of the most prevalent autoimmune diseases, with a female-to-male ratio as high as 20:1-9:1^(7)^. The involvement of lacrimal and salivary glands results in the typical features of dry eye and xerostomia. The symptoms are often highly variable and can progress slowly, making timely diagnosis a challenging issue^(1,8)^. A delayed diagnosis compromises early treatment, leading to potentially serious consequences that affect the patient’s quality of life, pose socioeconomic burden, and have life-threatening sequelae^(9)^. The clinical work-up typically involves a variety of tests, including tear and salivary function tests, serological autoantibody biomarkers, salivary gland biopsy, and systemic endocrine findings^(6)^.

The search for biomarkers is a convenient and non-invasive tool for the diagnosis of SS. Traditional serum biomarkers include SS-A/Ro, SS-B/La, antinuclear antibody (ANA), and rheumatoid factor (RF)^(7)^. Although important for the diagnosis of SS, they are not always positive in the patients, especially during the early stages of the disease. Furthermore, SS-A/Ro and SS-B/La are positive only in half of the patients with SS who have dry eye symptoms^(7,10)^.

Numerous studies have differentiated SS from dry eye and control populations based on variations in tear film protein expression^(11-13)^, implying that biomarker profiling may be of significant value in dry eye diagnosis^(14,15)^.

Many studies have suggested that quantification of a single biomarker such as lysozyme^(16)^, lipocalin^(12)^, or lactoferrin^(11,13,15,16)^ can replace detailed biochemical profiling and could serve as a supplemental diagnostic parameter along with traditional assessments of the ocular surface. Studies that investigate the lysozyme present within the tear film of different subgroups of dry eye patients point to potential differences in lacrimal gland function, thus offering more options for accurate diagnosis and treatment.

This study aimed to evaluate a new inexpensive and non-invasive biomarker for the screening of Sjögren’s syndrome dry eye. To this end, we evaluated the concentration of tear lysozyme in individuals with Sjögren’s syndrome (SS), meibomian gland dysfunction (MGD), and non-dry-eye disease (NDED).

## METHODS

### Study design

Ninety women were recruited: 30 SS, 30 MGD, and 30 NDED healthy women.

The inclusion criteria were: age >18 years and a best corrected visual acuity (BCVA) of >20/30 for all subjects. For the SS group: women who met the Sjögren’s International Collaborative Clinical Alliance (SICCA) classification criteria for the diagnosis of SS^(6)^. For the MGD group: women who fulfilled the criteria for the diagnosis of MGD according to the International Workshop on MGD Diagnosis^(17)^. Control group: no signs of ocular surface disease.

The exclusion criteria were: chronic illnesses, smoking, history of contact lens use, ophthalmic surgery, preexisting ophthalmic conditions such as allergic conjunctivitis, uveitis, high myopia, lagophthalmos, any systemic diseases (such as diabetes mellitus or depression), and the use of any systemic medication.

The research protocol was approved by the Ethics Committee of the Institution, and all subjects gave their informed consent before being enrolled in the study.

### Ocular Surface Disease Index (OSDI)

All subjects were evaluated using the OSDI questionnaire^(18)^ translated into Spanish. The questionnaire contained 12 questions to aid in the assessment of the presence or absence of ocular dryness, irritation, heaviness, fatigue, and itching over the past 7 days. The total OSDI score was then calculated using the following formula: OSDI = (sum of scores for all questions answered) × 100/(total number of questions answered) × 4]. The test scale ranged from 0 to 100, with higher scores representing greater disability. The subjective discomfort symptoms were graded on the basis of the dry eye discomfort symptoms questionnaire (OSDI) scores as follows: 0-12 (no disability), 13-22 (light dry eye), 23-32 (moderate dry eye), and 33-100 (severe dry eye)^(18)^.

### Schirmer I test

Schirmer I test (without anesthesia) was performed before any drops were instilled into the eye. Standardized Schirmer strips were bent at the notch and placed carefully over the lower lid margin as far toward the temporal angle of the lids as possible. The patient was instructed to keep his or her eyelids closed during the test. The strips remained in place for 5 min or until complete saturation with tears, whichever was the earliest. Subsequently, the wetting of the strips was measured using the millimeter scale.

### Tear break-up time

Tear break-up time (TBUT) was measured by instilling 5 mL of 2% sodium fluorescein into the bulbar conjunctiva using a micropipette. Within 30 s, the patient was asked to stare straight ahead without blinking. TBUT was estimated by measuring the time elapsed from the last complete blink to the appearance of the first dry spot in the fluorescein-stained tear film without touching the eyelid^(19)^.

### Vital staining

Conjunctival lissamine green staining was performed using lissamine green strips (Diagnóstico Ocular, Buenos Aires, Argentina) dampened with 0.9% sodium chloride and gently applied to the inferior fornix. The staining pattern was evaluated and graded according to the ocular Sjögren’s International Collaborative Clinical Alliance grading score^(20)^.

### Impression cytology

Impression cytology was used to obtain samples from the superficial epithelial cell layers of the inferior tarsal conjunctiva. Semicircular filters, approximately 15 mm in diameter (cellulose ester filter 22-mm pore; Millipore Corp., Bedford, MA, USA), were applied to the inferior tarsal conjunctiva after instilling a drop of topical anesthetic (proparacaine hydrochloride ophthalmic solution 0.5%) in each eye, and the excess fluid was wiped away. The paper fragments were applied for approximately 10 s, and after applying gentle pressure with the blunt end of the forceps, the fragments were peeled off and immediately immersed in tubes containing absolute ethanol. After fixation, the specimens were rehydrated in 70% ethyl alcohol and placed successively in periodic acid-Schiff reagent, sodium metabisulfite, Gill’s hematoxylin, and Scott’s tap water. The specimens were then rinsed with 95% alcohol and absolute alcohol. Xylene was used to make the filter paper transparent. Before mounting, the filter paper was placed with the epithelial cells facing up. In each sample, the degree of Nelson was determined by considering the density, morphology, cytoplasmic staining affinity, and the nucleus/cytoplasm relationship of the epithelial and conjunctival goblet cells. According to this system, 4 stages (0-3) can be distinguished, with 0 and 1 being normal and 2 and 3 referring to an altered state^(21)^.

### Tear lysozyme concentration

The tears were collected by gently applying a 5-mm-diameter filter paper disc in the inferior conjunctival cul-de-sac of both eyes for 1 min, with the eyes closed. The samples were stored at -20°C until they were processed. To determine tear lysozyme concentration, we performed the *Micrococcus lysodeikticus* (ATCC 4698, 770; Sigma-Aldrich, St. Louis, MO) agar diffusion assay in Mueller Hinton agar plates (Bio Merieux, Marcy l’Etoile, France). Each disc was placed on the plate containing the *Micrococcus lysodeikticus* (2 × 106 CFU/mL) suspension gel, and the inhibition halo was measured after 24 h. To calculate the lysozyme concentration, a standard curve was obtained using identical discs dampened with 10000, 1000, 100, and 10 mg/mL of lysozyme (ATCC 4698, L6876; Sigma-Aldrich) diluted in phosphate-buffered saline (Invitrogen Corp., Carlsbad, CA). Values £1000 mg/mL were considered abnormal^(22)^.

### Statistical analysis

The variables evaluated were OSDI, the Schirmer 1 test, TBUT, conjunctival green lissamine staining, conjunctival impression cytology, and tear lysozyme. All data were analyzed using the statistical software SPSS 17.0 (WinWrap Basic, Copyright 1993-2007 Polar Engineering and Consulting) according to the following procedure: the Levene’s test was initially performed, and ANOVA was done when the p value was >0.05. If the ANOVA results were significant (p£0.05), the Bonferroni post-hoc test (a=0.05) was further used for multiple comparisons. When there was no significant difference in the ANOVA results (p>0.05), the statistical tests were ended.

## RESULTS

The performance of the parameters for SS, MGD, and NDED is summarized in table 1.

**Table 1:** Ocular questionnaire, clinical test, impression cytology, and tear lysozyme

Variable	SS (n=30)	MGD (n=30)	NDED (n=30)	*p*-value
OSDI, total	64.5 ± 22.6	43.5 ± 21.4	6.7 ± 4.3	0.02^*^
Schirmer I test, mm/5 min	4.95 ± 2.25	13.28 ± 1.53	13.70 ± 1.39	<0.01^**^
TBUT, s	3.97 ± 1.47	3.95 ± 0.86	7.25 ± 1.90	<0.01^***^
Lissamine	6.18 ± 2.14	5.27 ± 1.27	1.52 ± 0.97	<0.01^***^
green, score				
Impression	1.88 ± 0.92	1.67 ± 0.56	0.45 ± 0.44	<0.01^***^
cytology, score				
Tear lysozyme, µg/mL	751.25 ± 244.73	1423.67 ± 182.75	1409.90 ± 188.21	<0.01^**^

All continuous variables are presented as mean ± standard deviation.

NDE= non-dry eye; SS= Sjögren´s syndrome; DED= dry eye disease; MGD= meibomian gland dysfunction; OSDI= ocular surface disease index; TBUT= tear breakup time.

* One-way ANOVA, Bonferroni post-hoc multiple comparison between groups, p<0.05;

** Statistically significant for SS vs NDED and SS vs MGD;

*** Statistically significant for SS vs NDED and MGD vs NDED.

All were women, and no differences were noted between the groups in terms of age (SS: 54 ± 9; MGD: 52 ± 12, and NDED 55 ± 14).

The total score of the OSDI questionnaire was different among the groups, showing a lower score for NDED, an average score for MGD patients, and a higher score for SS patients.

TBUT, lissamine green vital staining, and impression cytology did not reveal any differences between the SS and MGD patients.

On the other hand, both the Schirmer test and the tear lysozyme test indicated differences between the SS and MGD patients. In both the tests, the values obtained for the MGD patients were similar to those obtained for NDED.

All SS patients presented tear lysozyme values <1000 mg/mL; in sharp contrast, the MGD and NDED subjects presented values >1000 mg/mL.

Impression cytology scores were found to be poor in discriminating between the SS and non-SS DED groups under comparison.

## DISCUSSION

The OSDI is a frequently used questionnaire that consists of 12 questions probing dry eye symptoms. This questionnaire is used to distinguish between normal subjects and those with dry eye and can be employed to measure the severity of the eye symptoms as well as their effect on visual function. Dry eye patients often complain that it is difficult for them to see and that they face vision disturbances despite having a good visual acuity. Such issues correspond to a low tear film surface quality^(1)^.

In our study, OSDI was significantly different between the groups; however, several authors have shown that OSDI is a method to evaluate the severity of the dry eye and that it does not help in differentiating between SS and MGD^(18)^.

The data from this study assert that the tear lysozyme concentration is low in patients with SS dry eye disease but not in those with MGD dry eye disease. This finding alludes the predictive capability of the tear lysozyme concentration in diagnosing SS dry eye disease.

The healthy lachrymal gland secretes a complex fluid that contains proteins, nutrients, hormones, growth factors, and immunoglobulins in an isotonic electrolyte solution. Lachrymal gland function impairment in SS is also caused by lymphocytic infiltration^(23)^.

Tears contain several molecules with antimicrobial activity that can directly kill or prevent the growth of a range of pathogenic organisms.

It is remarkable in this context that the tear protein lactoferrin and lysozyme are localized in the secretory granules of the lachrymal gland, and it is believed they are most likely secreted together. This observation suggests that they could act as indicators of lachrymal gland functioning^(24-26)^. Both lactoferrin and lysozyme are crucial for protecting the ocular surface^(24-26)^.

Lysozyme is secreted by the main and accessory lacrimal glands, and it accounts for up to 20%-30% of the total proteins in both basal and reflex tears. Lysozyme catalyzes the hydrolysis of 1,4-beta-linkages between N-acetylmuramic acid and N-acetyl-D-glucosamine in the peptidoglycan backbone of the bacterial cell wall. The compromised cell wall is no longer able to maintain a stable osmotic environment, and cell lysis ensues^(27)^.

A low level of tear lysozyme implies a reduced bacteriostatic effect of the tears. This could explain why patients with dry eye disease and Sjögren’s syndrome are more prone to ocular surface infections than those with dry eyes caused by an alteration of the meibomian glands.

In conclusion, reduced tear lysozyme concentration showed an excellent diagnostic performance in distinguishing patients affected by Sjögren´s syndrome from those with non-dry-eye or MGD dry eye. This study has established that tear lysozyme could be considered as a promising simple, inexpensive, and non-invasive biomarker with a high accuracy for diagnosing Sjögren´s syndrome.
